# Chinese expert consensus for neurological monitoring and long-term follow-up in neonatal patients supported on extracorporeal membrane oxygenation (ECMO) (2025): a protocol

**DOI:** 10.3389/fmed.2026.1775437

**Published:** 2026-03-25

**Authors:** Meizhu Lu, Jiao Li, Shouliang Jiang, Jing Shi, Yinzi Helan

**Affiliations:** 1 Department of Pediatrics, West China Second University Hospital, Sichuan University, Chengdu, China; 2 Key Laboratory of Birth Defects and Related Diseases of Women and Children (Sichuan University), Ministry of Education, Chengdu, Sichuan, China; 3 Department of Neonatology Nursing, West China Second University Hospital, Sichuan University, Chengdu, China

**Keywords:** ECMO, expert consensus, extracorporeal membrane oxygenation, neonate, neurological monitoring and follow-up protocol

## Abstract

**Introduction:**

Neurological complications are a major contributor to morbidity in neonatal patients supported with extracorporeal membrane oxygenation (ECMO). Adequate neurological monitoring during ECMO enables the early recognition and management of neurological injury. In addition, long-term neurodevelopmental follow-up after ECMO allows for the early identification of neurodevelopmental delay and the timely initiation of interventions. Therefore, standardized neurological monitoring and structured long-term follow-up in neonatal patients receiving ECMO support are essential to reduce the incidence and severity of neurological complications, improve prognosis, and enhance the quality of life of affected children.

**Methods and analysis:**

This is a Protocol for a Systematic Review and GRADE-Based Expert Consensus. This protocol outlines the methodology and process for developing the “Chinese Expert Consensus for Neurological Monitoring and Long-term Follow-Up in Neonatal Patients Supported on extracorporeal membrane oxygenation (ECMO) (2025).” It details the purpose, target patient population, composition of the consensus working group, presentation and collection of clinical questions, rigorous evaluation and summarization of evidence, and formulation of expert recommendations. This structured approach enhances the standardization and transparency of the consensus development process.

**Ethics and dissemination:**

This expert consensus has been registered bilingually with the International Practice Guidelines Registry Platform (IPGRP) under the registration number PREPARE-2024CN1051. The final recommendations will be disseminated through publication in peer-reviewed journals, presentations at national and international conferences, educational workshops, and postings on widely used medical platforms in China. The aim is to support healthcare professionals, improve clinical outcomes, and inform health policy development in neonatal critical care.

## 1 Introduction

Extracorporeal membrane oxygenation (ECMO) is an advanced life-support technique used to rescue newborns with severe cardiopulmonary failure when conventional medical interventions have failed (1). According to the latest 2022 data from the Extracorporeal Life Support Organization (ELSO), ECMO for neonatal respiratory disease is associated with the highest survival rates compared with adults and children, with 24-h survival after cannulation and survival to hospital discharge of 83% and 69%, respectively (2). For neonatal heart disease and neonatal extracorporeal cardiopulmonary resuscitation (ECPR), the corresponding 24-h post-cannulation survival and discharge survival rates are 72% and 48%, 68% and 44%, respectively. As the application of ECMO in neonatal critical care expands, neurological complications among surviving infants have emerged as a clinical focus.

The reported incidence of neurological complications in neonates supported with ECMO ranges from 17.3% to 52% (3–7), whose variation may be associated with gestational age, ECMO mode, and the intensity of imaging screening, making these complications a leading cause of morbidity and mortality in this population (4). The immaturity of the neonatal brain renders it particularly vulnerable to injury during ECMO support. In addition, several ECMO-related mechanical risk factors—such as systemic inflammatory responses induced by blood exposure to the surface of artificial blood vessels, altered blood flow patterns, unilateral cannulation of the carotid artery and internal jugular vein, and systemic heparinization during ECMO—may further impair the blood-brain barrier and exacerbate hemorrhagic or ischemic injuries (5, 8). Neurological complications in ECMO-supported neonates include acute-phase injuries, such as intracranial hemorrhage, hypoxic–ischemic brain injury, acute cerebral infarction, and cerebral edema (3, 8), as well as long-term sequelae, including motor deficits, cognitive delay, and behavioral abnormalities. These complications substantially affect both survival and long-term quality of life (9). Timely neurological monitoring during ECMO enables early recognition and management of neurological injury, while structured long-term neurodevelopmental follow-up facilitates the early detection and intervention of neurodevelopmental delays. Therefore, comprehensive neuromonitoring and long-term follow-up of ECMO-supported neonates are essential to reduce neurological complications and mortality and to improve long-term outcomes.

Through a systematic review and meta-analysis, the research team identified that neonates undergoing ECMO are associated with a variety of neuropsychological complications, and highlighted the necessity of conducting neurological function monitoring and long-term follow-up for this cohort of neonates (10). The ELSO has published guidelines on long-term follow-up (September 2021) and routine neuromonitoring (October 2023) for neonatal and pediatric patients supported with ECMO (8, 11). In its long-term follow-up guidelines, ELSO emphasizes that the availability of international, national, and local resources, as well as variations in the organization of primary, secondary, and tertiary healthcare services, may influence the feasibility of implementing follow-up programs. This underscores the need for guidance tailored to national contexts. However, to date, neuro-monitoring modalities for neonates receiving ECMO support are diverse yet lack standardization in China. Advanced devices such as near-infrared spectroscopy (NIRS) are being gradually adopted in tertiary medical centers, but their accessibility remains limited in primary and secondary hospitals. Additionally, the long-term neurodevelopmental follow-up system is relatively underdeveloped, with a lack of standardized assessment protocols and intervention pathways, thereby affecting the accessibility and consistency of care and outcomes.

To address this gap, We initiated and formulated the *Chinese Expert Consensus for Neurological Monitoring and Long-term Follow-Up in Neonatal Patients Supported on Extracorporeal Membrane Oxygenation (ECMO) (2025)* (hereinafter referred to as *the Consensus*). Based on the latest domestic and international research findings, this consensus fully addresses the core issues regarding neuromonitoring and long-term follow-up for ECMO-supported neonates from the perspective of clinical practice and in combination with China’s national context. Using the Delphi method, we finally developed an evidence-based expert consensus integrated with the insights of the expert panel members. Its overall objective is to standardize the clinical practice of neuromonitoring and long-term follow-up for neonates receiving ECMO support, reduce the incidence and mortality of neurological complications, and improve long-term treatment outcomes. Potential benefits include enhanced parental satisfaction. This paper elaborates on the methodology and technical roadmap adopted for the development of this Consensus in detail. This expert consensus covers both VA and VV ECMO neonates.

## 2 Methods and analysis

### 2.1 Expert consensus development methodology

**FIGURE 1:**
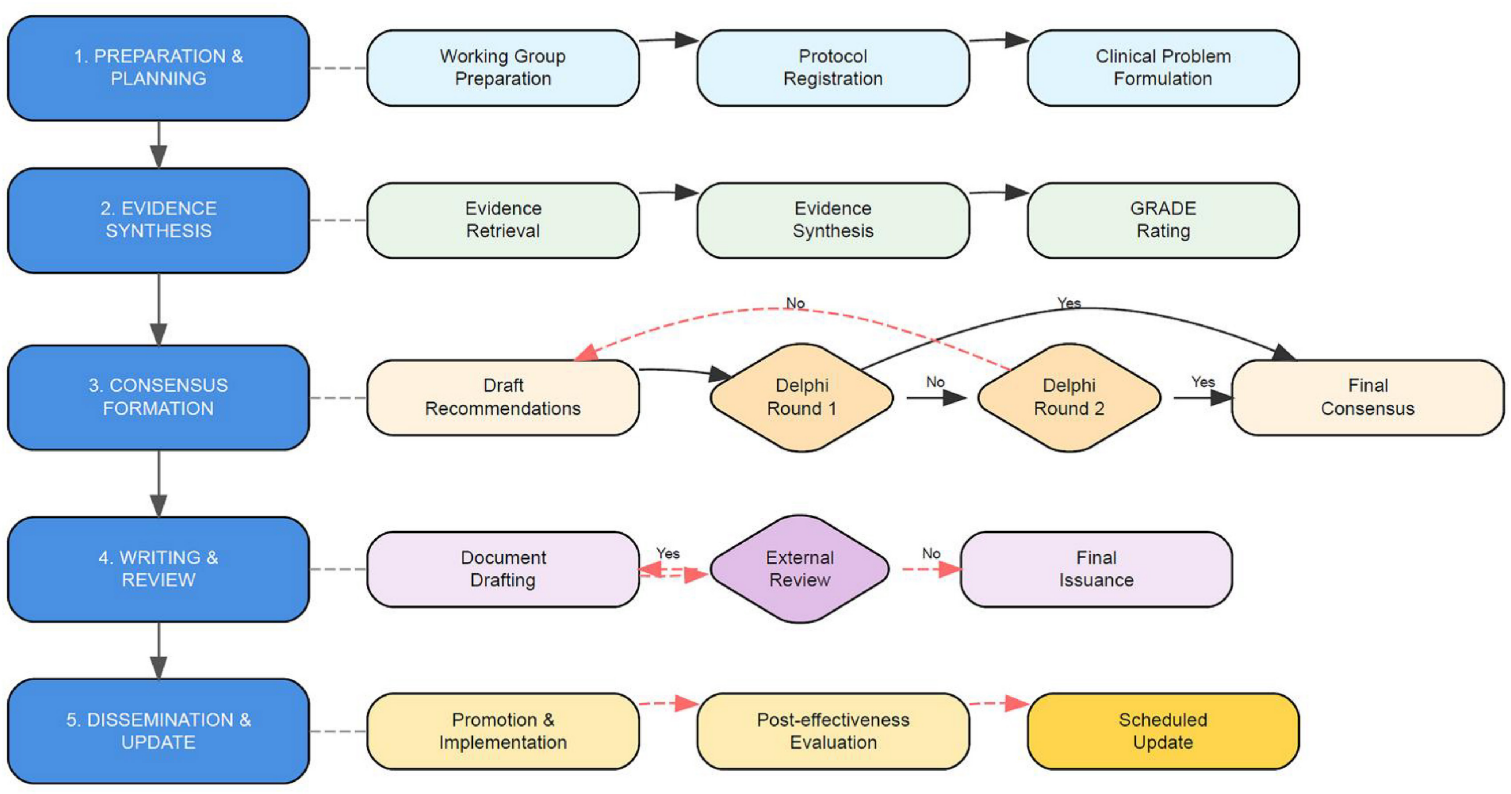
Overall flowchart for the development of expert consensus on neurological monitoring and long-term follow-up in neonatal extra corporeal membrane oxygenation (ECMO) patients.

The methodology and steps for developing this expert consensus were primarily based on the “World Health Organization Manual for Guideline Development (2014)” (12), “Guideline 2.0: A Comprehensive Inventory Systematically Developed for the Successful Development of Guidelines,” (13) “Basic Methods and Procedures for the Development/Revision of Clinical Guidelines” published by the Chinese Medical Association in 2016 (14), and “Guiding Principles for Developing/Revising Clinical Diagnosis and Treatment Guidelines in China (2022 Edition)” (15). Additional guidance was drawn from the “Appraisal of Guidelines for Research and Evaluation II (AGREE II)” (16), and “Reporting Items for Practice Guidelines in Healthcare (RIGHT)” (17). The development schedule is shown in Table 1. A visual Flowchart summarizing all the steps is illustrated in Figure 1.

**TABLE 1:** Schedule, leading teams and work contents for the development of expert consensus.

Stage	Timeline	Leading team	Work content
Project initiation and preparation	Oct 2024	Steering committee	Consensus initiation and working group preparation
	Nov 2024	Secretariat	Protocol development and registration
Evidence synthesis	Jan 2025	Evidence evaluation group	Clinical problem formulation, collection and prioritization
	Mar–Jun 2025	Evidence evaluation group	Systematic evidence retrieval, critical appraisal and GRADE rating
Consensus formation	Aug–Nov 2025	Consensus panel	Two-round Delphi survey and expert feedback collection
	Nov 2025	Consensus panel	Final consensus meeting and recommendation grading
Writing and external review	Jan–May 2026	Secretariat	Drafting of expert consensus document
	May 2026	Steering committee and external reviewers	External review, revision and final approval
Dissemination and update	Jul 2026–2031	Steering committee and secretariat	Promotion, implementation, evaluation and scheduled update

### 2.2 Expert consensus-building bodies

This expert consensus was initiated and organized by the Second West China Hospital of Sichuan University, which assumes the administrative and ethical responsibilities during the formulation of this consensus. The expert team for formulating the consensus consists of multidisciplinary experts assembled under the leadership of the aforementioned institution.

### 2.3 Working group on expert consensus

Referring to the recommendations for the development of clinical practice guidelines (18), the working group included a chief expert, steering committee, evidence evaluation group, consensus group for recommendations, external review group, and secretarial group. At least 60% of the working group members shall hold valid annual center certifications for neonatal ECMO cases, or possess equivalent qualifications recognized by national or international pediatric critical care academic organizations. To cover the clinical practice characteristics and regional medical resource differences in neonatal ECMO, the working group shall include representatives from all major geographic regions of the country, and each major geographic region shall have at least two votes in the consensus voting process. The composition and specific responsibilities of the working group were as follows.

The chief experts included two individuals: one chief clinical expert and one chief methodology expert. The chief clinical expert served as the overall leader of the expert consensus, had decision-making authority over all stages of expert consensus development, was responsible for reviewing and writing the final manuscript of the expert consensus, and ensured the practicality of the clinical system. The chief methodology expert provided methodological guidance and training and carried out quality control throughout the development process.

The steering committee consisted of seven experts from different disciplines, including one clinical expert in neonatology, one clinical expert in intensive care, one expert in pediatric neurology, one expert in pediatric rehparenttabilitation science, one expert in evidence-based medicine, one expert in methodology for guideline development, and one expert in management. The main responsibilities of the steering committee included: (i) determining the purpose of the consensus, target population, scope of application, and process methodology; (ii) guiding the drafting and review of the expert consensus plan; (iii) identifying and forming the working groups and clarifying the responsibilities of each group; (iv) reviewing and obtaining declarations of interest and addressing any conflicts of interest among potential working group members, including disqualifying members from voting directly, requiring relevant experts to recuse themselves from discussions or voting on specific issues, or attaching disclosures alongside their comments. All processes and outcomes of such management shall be documented and made publicly available; (v) organizing consensus-related meetings on a regular basis; (vi) reviewing and determining the population, intervention, control, and outcome (PICO) format clinical questions, identifying outcome metrics, and evaluating the quality of evidence; (vii) overseeing and guiding the consensus development process; (viii) guiding the drafting, review, and approval of recommendations; (ix) submitting the expert consensus, determining layout, proofreading, and publication; and (x) approving the expert consensus for publication and promoting its dissemination and updating.

The Evidence Evaluation Group consisted of 5–8 researchers experienced in evidence retrieval and evaluation, with backgrounds in neonatal medicine, intensive care medicine, pediatric neurology and rehabilitation medicine, and evidence-based medicine. Their main responsibilities included: (i) literature search and screening of evidence; (ii) evidence evaluation and synthesis; (iii) GRADE evidence grading, writing evidence summary tables, and developing recommendations; and (iv) writing the first draft of the expert consensus, forming the final draft after modification based on expert feedback, and submitting it to the steering committee for review.

The consensus expert group consisted of 15–20 multidisciplinary clinical, medical, and basic research experts. Their main responsibilities were as follows: (i) identifying clinical problems; (ii) voting and reaching consensus on recommended opinions; and (iii) finalizing the full text of the expert consensus.

The external review panel comprised five peer experts who were not involved in the development of the expert consensus. Their main responsibility was to review the final version of the expert consensus and its recommendations formed by the consensus group.

The secretarial group consisted of three personnel, including one clinical researcher in neonatal medicine, one researcher in intensive care medicine, and one researcher in guideline consensus methodology. Their main responsibilities included: (i) writing the consensus plan and completing the consensus registration; (ii) researching clinical issues according to the PICO principles; (iii) organizing consensus meetings for recommended opinions; (iv) documenting the entire process of expert consensus development in detail; and (v) submitting the expert consensus.

Parent representatives were involved in both the design phase and implementation strategy development. During the design phase, two parent representatives randomly selected from the hospital’s Parent Advisory Committee (parents of children who underwent ECMO and were discharged ≥ 12 months ago) contributed to identifying family priorities that informed clinical question formulation. During implementation strategy development, they provided feedback on feasibility and resource allocation. Their participation was limited to discussions; they had no voting rights regarding evidence grading or final recommendation formulation.

### 2.4 Registration of expert consensus

This expert consensus has been registered bilingually with the International Practice Guidelines Registry Platform under the registration number: PREPARE-2024CN1051.

### 2.5 Purpose and scope of the expert consensus

The proposed title of this consensus is *“Chinese Extracorporeal Membrane Oxygenation (ECMO)-Supported Neonatal Neuromonitoring and Long-Term Follow-Up Expert Consensus (2025).”* This expert consensus is intended to provide a reference for medical personnel involved in the management of neonatal ECMO support, to standardize neuromonitoring and long-term follow-up practices, and to reduce the incidence of neurological complications and mortality among ECMO-supported neonates. The target population for this consensus is critically ill neonates. The consensus is applicable to clinical staff in neonatal and critical care medicine, pediatric neurology, and rehabilitation medicine, as well as other medical, healthcare technology, and management personnel.

### 2.6 Clinical problem formulation, collection, and screening

Combined with the characteristics of neonatal patients on ECMO, the main issues related to neuromonitoring and long-term follow-up were identified. These issues were gathered through multiple channels: the Evidence Evaluation Group conducted a literature search to summarize controversial and unresolved topics; multidisciplinary experts in neonatology, intensive care medicine, rehabilitation, and related fields convened a seminar to document problems encountered in clinical practice; a retrospective analysis of multicenter neonatal ECMO cases was performed to identify the high incidence of neurological complications and gaps in follow-up; and communication with patients’ families was conducted to understand their needs and deficiencies in long-term follow-up. The framework of the collected clinical questions was refined according to PICO principles. Then, the questions were scored by a consensus expert panel using a two-round Delphi method, based on clinical relevance, research evidence, actionability, and multidisciplinary value. A structured list of clinical questions was subsequently developed.

### 2.7 Literature search and evaluation of evidence

A literature search strategy was developed, and the following databases were searched: PubMed, Embase, Cochrane Library, Web of Science, CNKI, Wanfang Medical Network, MEDLINE, CBM, VIP, and the Joanna Briggs Institute’s Evidence-Based Practice Database. Guideline-related resources included the Scottish Intercollegiate Guidelines Network, International Guidelines Collaboration Network, Guidelines International Network, National Institute for Health and Care Excellence, and other relevant organizations and associations. Additional searches were conducted via Google Scholar and the references cited in the included studies. Relevant references were included in this study. The literature search was limited to publications up to 30 November 2024, in both English and Chinese. The search strategy combined MeSH terms and free-text keywords, developed based on the PICO framework and core clinical problems. (For detailed information regarding PICO, search strategy, English and Chinese MeSH terms, see Supplementary Document 1).

The retrieved literature was screened according to predefined eligibility criteria. Inclusion criteria encompassed clinical guidelines, expert consensus, systematic reviews and meta-analyses, randomized controlled trials, and observational studies. Exclusion criteria included unavailable full text, reports without references, translated guidelines, and literature with substandard quality evaluation results. Literature screening will be conducted independently by two researchers. First, an initial screening will be performed based on titles and abstracts, followed by a full-text review for secondary screening. The screening results from the two researchers will be compared to test for inter-rater consistency.

Data extraction will be completed independently by two researchers using a pre-designed data extraction form. The extracted information includes the first author, year of publication, study design, PICO elements, sample size, primary outcome measures, and result data. After completion of extraction, a cross-check will be conducted on the two extraction forms.

Evidence evaluation was performed using different tools depending on the type of literature. Clinical practice guidelines were evaluated using AGREE II, systematic reviews and meta-analyses were evaluated using AMSTAR 2 (19), randomized controlled trials were evaluated using the Cochrane Risk of Bias tool (ROB 2.0) (20), and cohort and case-control studies were evaluated using the Newcastle-Ottawa Scale (21). Any discrepancies arising during literature screening, data extraction and quality assessment shall first be resolved through discussion to reach a consensus between the two researchers; if the discrepancies persist, a third senior researcher will be consulted for a final decision.

### 2.8 Grading the quality of evidence

The GRADE methodology was adopted, using the GRADE manual as a guide and the GRADEpro/Guideline Development Tool^1^ to evaluate and grade the quality of the included evidence and develop a GRADE evidence summary table (22). When applying the GRADE approach, the initial quality of the evidence was determined and then categorized as “high, moderate, low, and very low” based on five downgrading factors (risk of bias, inconsistency, non-directness, imprecision, and publication bias) and three upgrading factors (large effect sizes, dose-response relationships, and exclusion of confounding factors).

### 2.9 Formation of recommendations

Based on the level of evidence, initial recommendations were formulated by integrating clinical experience, resource availability, and a balance of benefits and risks, with the strength of each recommendation (strong or weak) clearly specified. The draft recommendations were reviewed by members of the expert consensus group using the Delphi method. The rules for reaching a consensus are as follows: a recommendation shall be deemed as reaching a consensus if the proportion of experts participating in the consensus vote who approve of the recommendation is **≥**70%; for recommendations that fail to reach a consensus but have an approval rate of 50%–69%, they shall be revised based on expert opinions before entering the next round of expert consensus discussions; if the approval rate is <50%, the recommendation shall be deleted. Then, the final document, including the strength of recommendation, level of evidence, and scope of application, was finalized and validated by the steering committee. The secretarial team documented the formation process of the recommendations in detail to ensure that the source of evidence and the development process of each recommendation were fully traceable.

### 2.10 Expert consensus writing and external reviews

After the recommendations were completed, the secretarial and evidence evaluation groups prepared the first draft of the full text of the consensus. The draft was written in modules by designated personnel, followed by editing, integration, and internal review to produce the final version of the first draft. The drafting process was guided by AGREE II for guideline development and the International Practice Guideline Reporting Standards (RIGHT).

To solicit broader input from clinical medical staff and further assess the practicality of the consensus in clinical settings, an external review panel was invited to conduct an external review and provide feedback. The content of the consensus was subsequently revised and finalized based on the results of this review.

### 2.11 Approval and publication of expert consensus

The full text of the expert consensus was finalized and approved by the steering committee and published in a relevant field journal in 2026.

### 2.12 Dissemination and implementation of expert consensus

After the release of the expert consensus, the Neonatologists Branch of the Chinese Medical Doctors’ Association will promote the consensus through the following ways: (i) publishing it in authoritative journals; (ii) presenting it in domestic and international neonatal academic exchange conferences over the next 5 years; (iii) organizing special sessions for learning the consensus to ensure that clinical healthcare workers understand the guidelines; and (iv) posting interpretations of the clinical guideline to widely used medical websites in China, such as Ding Xiang Yuan, Medical Pulse, and Mace Medicine.

### 2.13 Update of the expert consensus

The expert consensus be continuously monitored, and any relevant evidence will be reviewed in a timely manner to determine whether an update is needed. Consensus updates will be considered under the following circumstances: (i) the emergence of new, important, and high-quality research findings, (ii) new high-quality evidence that challenges existing recommendations and suggests modifications and updates, and (iii) new high-quality evidence that suggests changes to existing recommendations in terms of their safety profiles, target populations, and precautions. This consensus is expected to be updated every 5 years.

## 3 Discussion

Extra corporeal membrane oxygenation is an advanced life-support technique widely used to treat neonates with severe cardiopulmonary failure, both significantly improving resuscitation success and survival rates. However, these patients remain at high risk of neurological complications during and after ECMO support, which can seriously affect both short- and long-term prognosis. Real-time and accurate neuromonitoring during ECMO is critical for the prevention and management of brain injury. Additionally, long-term follow-up is essential for assessing neurodevelopment, cognitive function, motor skills, and behavioral outcomes, enabling timely interventions and improving quality of life.

The development of an expert consensus on neuromonitoring and long-term follow-up for neonates supported by ECMO provides clear guidance on the timing, monitoring tools, and indications for neuromonitoring. It promotes a multidisciplinary approach that integrates the expertise of neonatology, critical care, neurology, and rehabilitation, thereby improving the consistency and quality of neuromonitoring practices. By specifying time points and assessment tools for long-term follow-up, the consensus helps focus attention on key stages of neurodevelopment. Early identification of potential neurodevelopmental problems through structured follow-up facilitates timely interventions.

Moreover, the consensus aims to minimize unnecessary monitoring and follow-up while ensuring that high-risk neonates receive appropriate and timely supervision. It provides a scientific basis for medical institutions to formulate relevant policies and allocate monitoring equipment and follow-up resources effectively. This guidance supports primary care hospitals within a tiered referral system in standardizing neuromonitoring and follow-up procedures for neonatal patients undergoing ECMO, thereby improving overall medical standards.

However, we fully recognize the challenges that may arise in the development of this consensus.First, neonatal ECMO is a high-risk and low-incidence rescue technique. There is a lack of large-scale, high-quality studies for reference, and significant heterogeneity exists among the patient population, which to some extent affects the evidence level of the recommendations. The strategy adopted by the expert working group is to strictly adhere to an evidence grading and recommendation strength evaluation system. In the absence of high-level evidence, the group integrates the best available clinical research, pathophysiological principles, and the practical experience of multidisciplinary experts. Through structured discussions and two rounds of the Delphi method, a clinically actionable consensus has been achieved. Second, the application and interpretation of neuromonitoring technologies vary across different centers due to differences in equipment, personnel, and expertise, which may hinder standardized implementation. To address this, the consensus emphasizes the importance of multidisciplinary team collaboration and technical training, and recommends establishing remote consultation mechanisms in centers where conditions permit, so as to enhance the consistency and accuracy of monitoring.

The expert consensus on ECMO-supported neuromonitoring and long-term follow-up in neonates is of great significance. It not only provides scientific guidance for clinical diagnosis and treatment but also contributes to improved long-term outcomes for children and advances the field of neonatal critical care. Its release serves as a bridge connecting clinical practice, research, and health policy, providing sustained momentum for optimizing neonatal ECMO treatment and follow-up systems.

## Footnotes

1
https://gdt.gradepro.org/app/

## References

[1] Mok YH Lee JH Cheifetz IM. Neonatal extracorporeal membrane oxygenation: update on management strategies and long-term outcomes. *Adv Neonatal Care.* (2016) 16:26–36. 10.1097/ANC.0000000000000244 26808515

[2] Tonna JE Boonstra PS MacLaren G Paden M Brodie D Anders M et al. Extracorporeal life support organization registry international report 2022: 100,000 survivors. *ASAIO J.* (2024) 70:131–43. 10.1097/MAT.0000000000002128 38181413 PMC10962646

[3] Raets MM Dudink J Ijsselstijn H van Heijst AF Lequin MH Houmes RJ et al. Brain injury associated with neonatal extracorporeal membrane oxygenation in the Netherlands: a nationwide evaluation spanning two decades. *Pediatr Crit Care Med.* (2013) 14:884–92. 10.1097/PCC.0b013e3182a555ac 24121484

[4] Polito A Barrett CS Wypij D Rycus PT Netto R Cogo PE et al. Neurologic complications in neonates supported with extracorporeal membrane oxygenation. An analysis of ELSO registry data. *Intensive Care Med.* (2013) 39:1594–601. 10.1007/s00134-013-2985-x 23749154

[5] IJsselstijn H Hunfeld M Schiller RM Houmes RJ Hoskote A Tibboel D et al. Improving long-term outcomes after extracorporeal membrane oxygenation: from observational follow-up programs toward risk stratification. *Front Pediatr.* (2018) 6:177. 10.3389/fped.2018.00177 30013958 PMC6036288

[6] Young A Vorster L Riviello J Dinh D Shah R Erklauer J et al. Multimodal neuromonitoring modalities of neonatal patients on extracorporeal membrane oxygenation. *J Perinatol.* (2025): 10.1038/s41372-025-02385-z [Epub ahead of print]. 40781152

[7] Fox J Jenks CL Farhat A Li X Liu Y James E et al. EEG is a predictor of neuroimaging abnormalities in pediatric extracorporeal membrane oxygenation. *J Clin Med.* (2020) 9:2512. 10.3390/jcm9082512 32759731 PMC7463499

[8] Pandiyan P Cvetkovic M Antonini MV Shappley RKH Karmakar SA Raman L. Clinical guidelines for routine neuromonitoring in neonatal and pediatric patients supported on extracorporeal membrane oxygenation. *ASAIO J.* (2023) 69:895–900. 10.1097/MAT.0000000000001896 37603797

[9] Boyle K Felling R Yiu A Battarjee W Schwartz JM Salorio C et al. Neurologic outcomes after extracorporeal membrane oxygenation: a systematic review. *Pediatr Crit Care Med.* (2018) 19:760–6. 10.1097/PCC.0000000000001612 29894448 PMC6086744

[10] Jiang S Yan P Wang H Tang J Mu D. Long-term follow-up of neuropsychological complications in neonates undergoing extracorporeal membrane oxygenation: a systematic review and meta-analysis. *BMC Pediatr.* (2024) 24:77. 10.1186/s12887-024-04564-x 38267850 PMC10807126

[11] Ijsselstijn H Schiller RM Holder C Shappley RKH Wray J Hoskote A. Extracorporeal Life Support Organization (ELSO) guidelines for follow-up after neonatal and pediatric extracorporeal membrane oxygenation. *ASAIO J.* (2021) 67:955–63. 10.1097/MAT.0000000000001525 34324443

[12] WHO *WHO Handbook for Guideline Development.* 2nd ed. Geneva: WHO (2014).

[13] Schünemann JS Wiercioch W Etxeandia I Falavigna M Santesso N Mustafa R et al. Guidelines 2.0: a comprehensive checklist systematically developed for successful guideline development. *Chin J Evid-Based Med.* (2014) 14:1135–49. 10.1016/j.zefq.2021.01.009 34023244

[14] Jiang JM Zhan SY Jia XW Fang H Zuo L Gao RL. Basic methods and procedures for developing/revising clinical guidelines. *Chin Med J.* (2016) 96:250–3. 10.3760/cma.j.cn112137-20211228-02911

[15] Chen YL Yang KH Wang SQ Kang DY Zhan SY Wang JY et al. Guiding principles for the development/revision of clinical diagnosis and treatment guidelines in China (2022 edition). *Chin Med J.* (2022) 102:6. 10.3760/cma.j.cn112137-20211228-02911.

[16] Brouwers MC Kerkvliet K Spithoff K. The AGREE reporting checklist: a tool to improve reporting of clinical practice guidelines. *BMJ.* (2016) 352:i1152. 10.1136/bmj.i1152 26957104 PMC5118873

[17] Chen Y Yang K Marušic A Qaseem A Meerpohl JJ Flottorp S et al. A reporting tool for practice guidelines in health care: the RIGHT statement. *Ann Intern Med.* (2017) 166:128–32. 10.7326/M16-1565 27893062

[18] Chen YL Ma YF Zhou Q Zhou YF Lu LT Zhai SD et al. Who should be involved in the development of clinical practice guidelines? *Concord Med J.* (2019) 10:524–30. 10.3969/j.issn.1674-9081.2019.05.017

[19] Shea BJ Reeves BC Wells G Thuku M Hamel C Moran J et al. AMSTAR 2: a critical appraisal tool for systematic reviews that include randomised or non-randomised studies of healthcare interventions, or both. *BMJ.* (2017) 358:j4008. 10.1136/bmj.j4008 28935701 PMC5833365

[20] Sterne JAC Savović J Page MJ Elbers RG Blencowe NS Boutron I et al. RoB 2: a revised tool for assessing risk of bias in randomised trials. *BMJ.* (2019) 366:l4898. 10.1136/bmj.l4898 31462531

[21] Stang A. Critical evaluation of the Newcastle-Ottawa scale for the assessment of the quality of nonrandomized studies in meta-analyses. *Eur J Epidemiol.* (2010) 25:603–5. 10.1007/s10654-010-9491-z 20652370

[22] Morgano GP Mbuagbaw L Santesso N Xie F Brozek JL Siebert U et al. Defining decision thresholds for judgments on health benefits and harms using the Grading of Recommendations Assessment, Development and Evaluation (GRADE) Evidence to Decision (EtD) frameworks: a protocol for a randomised methodological study (GRADE-THRESHOLD). *BMJ Open.* (2022) 12:e053246. 10.1136/bmjopen-2021-053246 35273045 PMC8915269

