# Supplementary material for: Chinese expert consensus for neurological monitoring and long-term follow-up in neonatal patients supported on extracorporeal membrane oxygenation (ECMO) (2025): a protocol
Source: Front Med (Lausanne). 2026 Mar 25;13:1775437. doi: 10.3389/fmed.2026.1775437 (PMC13057394; doi:10.3389/fmed.2026.1775437)
Supplement: Supplementary file 1 [file Data_Sheet_1.docx]

**Search Strategy for each database**

| **The database** | **Search strategy** |
| --- | --- |
| PubMed | #1"infant, newborn"[MeSH Terms] #2"Extracorporeal Membrane Oxygenation"[MeSH Terms]  #3 #1 AND #2 #4"neurological monitoring OR brain monitoring OR neuromonitor* OR neurological monitor* OR brain monitor* "[Title/Abstract] #5"long term care OR follow up OR patient follow up OR long term OR long-term OR follow-up"[Title/Abstract]) #6 #4 AND #5 #7 #3 AND #6 |
| Embase | #1 'neonate'/exp OR 'newborn'/exp OR neonat* OR newborn* OR infant)  #2 'extracorporeal membrane oxygenation'/exp OR 'ecmo'/exp OR 'extracorporeal membrane oxygenation' OR ECMO)  #3 #1 AND #2 #4'neurologic examination'/exp OR 'brain monitoring'/exp OR neuromonitor* OR 'neurological monitor*' OR 'brain monitor*' #5 'long term care'/exp OR 'patient follow up'/exp OR 'long term' OR 'long-term' OR follow-up #6 #4 AND #5 #7 #3 AND #6 |
| Cochrane Library | (“neonatal” OR “newborn” OR “infant”) AND (“extracorporeal membrane oxygenation” OR ECMO) AND “neurological monitoring” AND “follow-up” |
| Web of Science | (Topic:“neonatal” OR “newborn” OR “infant”) AND (Topic:“extracorporeal membrane oxygenation” OR ECMO) AND “neurological monitoring” AND “follow-up” |
| CNKI | （主题：新生儿 OR 婴幼儿 OR 婴儿) AND (主题：体外膜肺氧合 OR ECMO) AND (主题：神经监测 OR 神经功能 OR 长期随访) |
| Wanfang Medical Network | （题名或关键词：新生儿 OR 婴幼儿 OR 婴儿) AND (题名或关键词：体外膜肺氧合 OR ECMO) AND (题名或关键词：神经监测 OR 神经功能 OR 长期随访) |
| MEDLINE | ("Extracorporeal Membrane Oxygenation"[Mesh] OR ECMO OR "extracorporeal life support") AND ("Infant, Newborn"[Mesh] OR neonat* OR newborn) AND ("Neurologic Monitoring"[Mesh] OR "nervous system monitoring" OR "brain monitoring") AND ("Long-Term Care"[Mesh] OR "long term follow-up" OR "patient outcome assessment") AND (consensus OR guideline OR "practice guideline"[Publication Type]) |
| CBM | #1 "体外膜肺氧合"[主题词] OR "ECMO"[常用字段:智能] #2 "婴儿，新生"[主题词] OR "新生儿"[常用字段:智能] #3 #1 AND #2 #4 "神经系统监测"[主题词] OR "神经监测"[常用字段:智能] OR "长期随访"[常用字段:智能] #5 #3 AND #4 |
| VIP | （题名或关键词：新生儿 OR 婴幼儿 OR 婴儿) AND (题名或关键词：体外膜肺氧合 OR ECMO) AND (题名或关键词：神经监测 OR 神经功能 OR 长期随访) |
| The Joanna Briggs Institute’s Evidence-Based Practice Database | (neonate* OR newborn* OR infant* OR baby OR babies) AND (Extracorporeal Membrane Oxygenation OR ECMO OR ECLS) AND (neurological monitor* OR neuromonitor* OR brain monitor*) AND (follow-up OR "long term" OR outcome* OR survivorship) AND (guideline* OR consensus* OR recommendation*) |
|  |  |
